# The Role of Microglia in Brain Metastases: Mechanisms and Strategies

**DOI:** 10.14336/AD.2023.0514

**Published:** 2024-02-01

**Authors:** Ying Feng, Xueqing Hu, Yingru Zhang, Yan Wang

**Affiliations:** ^1^Department of Medical Oncology, Shuguang Hospital, Shanghai University of Traditional Chinese Medicine, Shanghai 201203, China; ^2^Academy of Integrative Medicine, Shanghai University of Traditional Chinese Medicine, Shanghai 201203, China

**Keywords:** brain metastases, microglia, inflammation, microenvironment, immunotherapy, drugs, delivery system

## Abstract

Brain metastases and related complications are one of the major fatal factors in cancer. Patients with breast cancer, lung cancer, and melanoma are at a high risk of developing brain metastases. However, the mechanisms underlying the brain metastatic cascade remain poorly understood. Microglia, one of the major resident macrophages in the brain parenchyma, are involved in multiple processes associated with brain metastasis, including inflammation, angiogenesis, and immune modulation. They also closely interact with metastatic cancer cells, astrocytes, and other immune cells. Current therapeutic approaches against metastatic brain cancers, including small-molecule drugs, antibody-coupled drugs (ADCs), and immune-checkpoint inhibitors (ICIs), have compromised efficacy owing to the impermeability of the blood-brain barrier (BBB) and complex brain microenvironment. Targeting microglia is one of the strategies for treating metastatic brain cancer. In this review, we summarize the multifaceted roles of microglia in brain metastases and highlight them as potential targets for future therapeutic interventions.

## 1. Introduction

Brain metastasis is one of the leading causes of cancer-related deaths worldwide. It may cause many complications, including cognitive impairment, hydrocephalus, cerebral hernia, and encephalomeningitis, resulting in a poor quality of life and short life span. Lung cancer, breast cancer, and melanoma have been reported to have a higher risk of brain metastases than other types of cancers [1-3]. Brain metastases develop in 30%-50% of patients with non-small cell lung cancer (NSCLC), which severely affect their overall survival [4]. An increasing number of patients with breast cancer develop brain metastases, especially those with HER2-positive or triple-negative tumors [5]. Brain metastases from melanoma are also common and have a poor prognosis, leading to the death of 60%-70% of patients with melanoma [6]. Currently, effective therapies for metastatic brain cancer are particularly limited because of the impermeability of the blood-brain barrier (BBB) and the immunosuppressive state of the brain. Currently, the following five treatment options are available for brain metastases: chemotherapy, local or whole-brain radiotherapy, surgery, molecularly targeted agents, and immune checkpoint inhibitors (ICIs). However, none of these have achieved satisfactory efficacy with tolerable side effects, and the five-year survival rates remain low [7, 8]. Therefore, new strategies are needed to effectively treat brain metastases.

The BBB maintains the brain microenvironment under a tight control by regulating fluctuations in the chemical composition, transport of immune cells, and entry of pathogens and toxins [9, 10]. The central nervous system (CNS) has also been considered to be immune privileged, a concept that involves keeping adaptive immunity and inflammation under tight control. Microglia, which are resident macrophages of the brain parenchyma, are key elements in brain metastases, participating in immune responses and maintaining CNS homeostasis [11, 12]. Not only the different phenotypes of microglia themselves, but also their crosstalk with the surrounding cells, affect the colonization, proliferation, and migration of tumor cells [13]. Activated microglia are classically divided into two distinct phenotypes: the classic M1 state with anti-tumor effects and M2 state with pro-tumor effects [14, 15]. Crosstalk between microglia-astrocytes, microglia-other immune cells, and microglia-tumor cells can help circulating tumor cells (CTCs) to colonize the brain parenchyma [16]. Targeting microglia can potentially aid in treating brain metastases [17, 18].

In this review, we summarize the microglia-mediated reprogramming of brain microenvironment in cancer in terms of inflammation, angiogenesis, and immune modulation. We will also discuss the interactions between microglia and other cell types in the brain microenvironment as well as current drugs targeting microglia for the treatment of metastatic brain cancers.

## 2. Microglia-mediated reprogramming of brain microenvironment in cancer

Microglia control the fates of neural progenitors, astrocyte activation, neuronal homeostasis, and synaptogenesis [19]. They may undergo distinct morphological, molecular, and functional changes that create distinct biological states associated with the onset and progression of various diseases, including autism, brain tumors, depression, and neurodegenerative diseases [20-22].

Successful intracerebral colonization requires that CTCs in the bloodstream be able to arrest and extravasate into brain capillaries, and then survive in the brain parenchyma. The BBB separates peripheral blood circulation from the tightly regulated CNS environment, and penetration of the BBB is the first step for CTCs to colonize the brain [23]. In addition to endothelial cells, astrocytes and pericytes are important components of the BBB [24]. Disruption of the BBB and the recruitment of peripheral immune cells are associated with CNS inflammation. Mechanistically, breast cancer cells pass through the BBB by overexpressing COX-2, HBEGF, and ST6GALNAC5. ST6GALNAC5 enhances the attachment of breast cancer cells to brain endothelial cells [25]. In addition to breast cancer, an antagonist of the HBEGF receptor was observed to reduce brain metastasis in NSCLC [26]. CXCL12-CXCR4 also promotes the invasion of breast cancer cells by increasing vascular permeability [27].

### 2.1 Inflammatory responses

**Figure 1.**
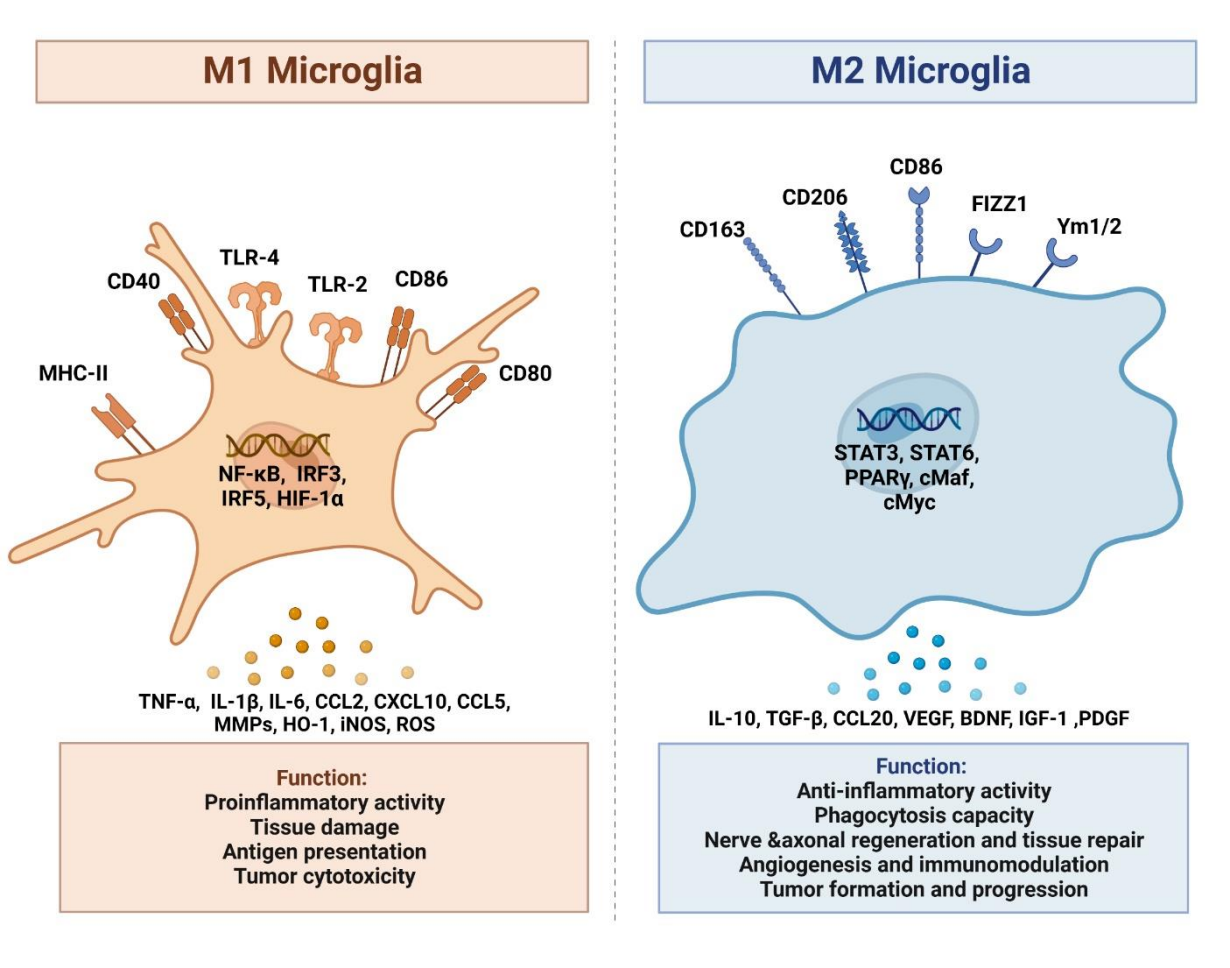
**Different functions of M1/M2 microglial phenotypes in brain microenvironment**. The figure illustrates that the resting microglia can be activated into M1/M2 phenotype in response to different stimuli. Microglia of M1 phenotype play roles in promoting inflammation, damaging the tissues, antigen presentation, and killing tumor cells. Microglia of M2 phenotype play roles in anti-inflammation, repairing of the tissues, promoting angiogenesis, suppressing immunity, and promoting tumor progression.

Primary tumors release a range of inflammatory factors, including tumor necrosis factor-alpha (TNF-α), interleukin 6 (IL-6), and interleukin 1-beta (IL-1β), which disrupt the BBB and cause inflammation in the CNS [28]. Under these conditions, microglia sense and respond to pro-inflammatory cytokines and modulate the responses of neighboring cells throughout the CNS [29]. Different stimuli in the microenvironment affect the differentiation of microglia into different subtypes [30]. The M1 phenotype (inducible nitric oxide synthase (iNOS) and CD86 markers) is pro-inflammatory, is mainly induced by lipopolysaccharide (LPS) or interferon-γ (IFN-γ), and secretes a large number of pro-inflammatory cytokines, such as IFN-γ, TNF-α, IL-1β, chemokines, such as CCL2, CXCL9, and CXCL10, protein hydrolases (heme oxygenase-1 (HO-1)), iNOS, and reactive oxygen species (ROS). M1 microglia play a key role in killing cancer cells [31]. He *et al.* have reported that LPS-activated M1 microglia induced apoptosis of metastatic lung cancer cells in the brain in a dose- and time-dependent manner *in vitro* [32]. In contrast, M2 microglia (CD206 and Arg-1 markers) can promote tumor growth by releasing various anti-inflammatory and immunosuppressive factors such as transforming growth factor-beta (TGF-β), IL-10, and CCL20 [33]. The M2 phenotype is induced by IL-4/IL-3 and is subdivided into M2a, M2b, and M2c phenotypes. A few surface markers, including CD206, CD163, CD86, FIZZ1, YM1/2, and arginase-1(Arg-1), can distinguish M2 from M1 microglia [34]. Consistently, patients with glioma with increased M2 microglial levels have a poor prognosis [35]. Massive infiltration of M2 microglia has also been reported in brain metastases of premenopausal patients with breast cancer [36]. The functional differences between M1 and M2 microglia are shown in Figure 1.

One mechanism of M1/M2 polarization is that the JAK2/STAT3 axis skews microglia/macrophage toward M2 polarization during cerebral ischemia/reperfusion injury [37]. In addition, STAT3 is a key checkpoint for suppressing anti-tumor immune responses [38]. IL-6 in brain metastatic NSCLC cells was observed to induce anti-inflammatory microglia via JAK2/STAT3 signaling, which in turn promoted the colonization of NSCLC cells [39]. Therefore, targeting the M1/M2 phenotypic polarization is one of the strategy to combat cancers with brain metastases.

Microglia can be divided into subpopulations based on their gene expression profiles. The naïve microglial population is characterized by high expression of microglial homeostasis genes (*Cx3cr1*, *Hexb*, and *Jun*). Microglial cluster in a primed state is marked by a high expression of mitochondrial genes and a slight decrease in the expression of microglial homeostasis genes. Microglial clusters in different inflammatory states are characterized by the upregulation of pro-inflammatory genes (*S100a4*, *S100a6*, and *S100a10*), anti-inflammatory genes (*Lgals1*, *Lgals3*, and *Ifitm3*), and migratory genes (*Vim* and *Anxa*), and downregulation of microglia homeostasis genes (*Cx3cr1*, *P2ry12*, *Hexb*, and *Cst3*) in myeloid cells in the CNS [40]. The classification of microglia based on their transcriptional status and their relationship with disease progression needs to be confirmed in future.

### 2.2 Immune modulation

Microglia are involved in both the innate and acquired immune responses. Phagocytosis is an important process by which microglia eradicate tumor cells. Amyloid beta (Aβ) secreted by melanoma cells suppresses microglial activation and prevents their phagocytosis [41]. Microglia of the M1 phenotype also function as antigen presenting cells that present antigens (MHC-II) to Th1 cells once activated, inducing T cell-mediated cell lysis and CD8^+^ T cell proliferation [42]. M2 microglia can help tumor cells establish a tumor-immunosuppressive environment and promote tumor progression [43].

**Figure 2.**
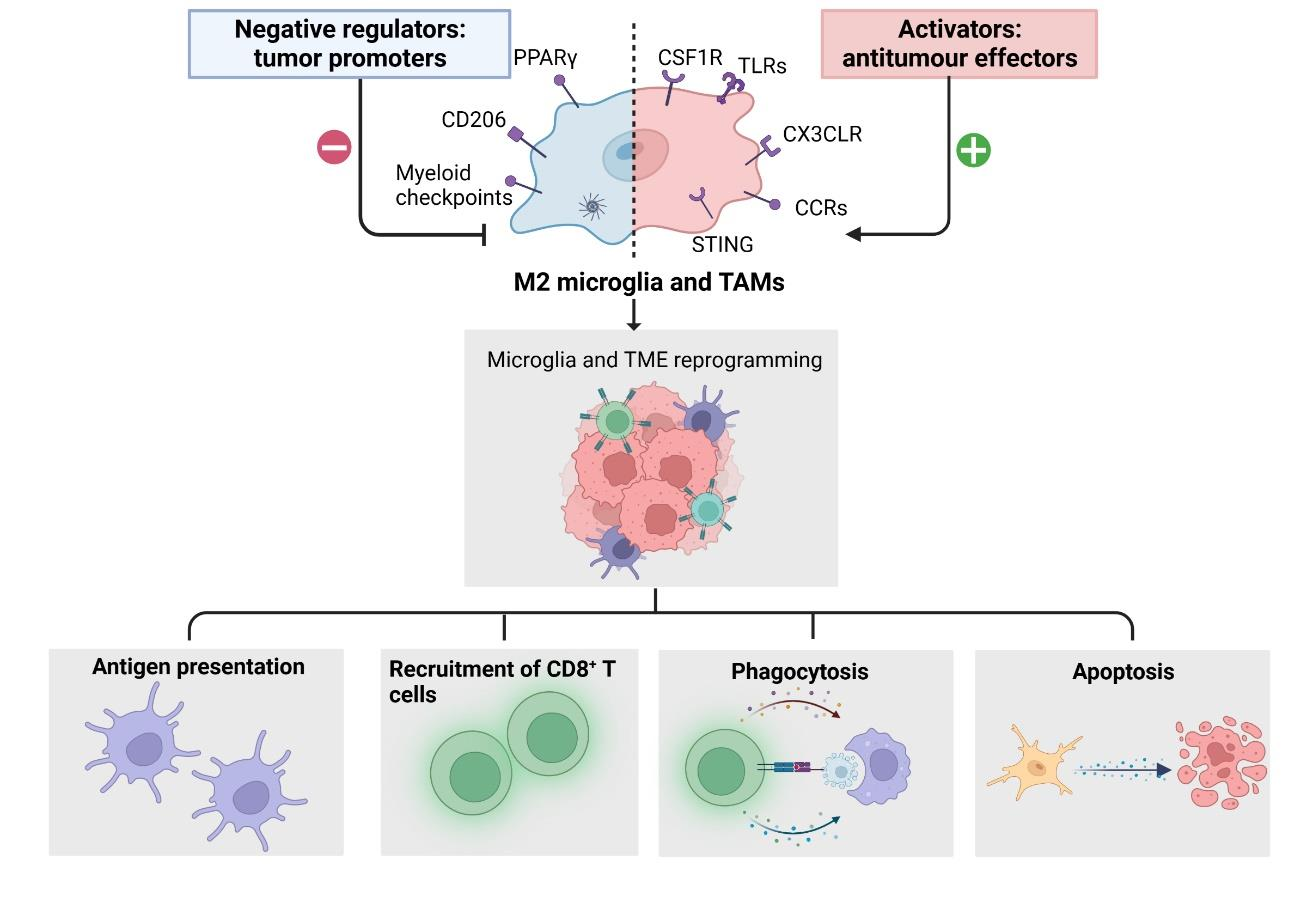
**Reprogramming of microglia and activation of innate and adaptive immune responses**. By inhibiting CSF1R, TLRs, CX3CLR, CCRs, and myeloid checkpoints, pro-inflammatory microglia can be activated to modulate innate immune responses, thereby enhancing antigen presentation, recruitment of CD8^+^T cells, and phagocytosis.

Nicotine promotes tumor progression by skewing the polarity of microglia toward the M2 phenotype and suppressing their anti-tumor innate immunity [44]. Mechanistically, nicotine polarizes microglia via the nAch receptor-STAT3 pathway and restricts their phagocytic ability through the overexpression of signal regulatory protein alpha (SIRPα) in microglia [44]. Native myeloid cells in the CNS include microglia and border-associated macrophages (BAMs). Guldner *et al.* have reported that *Cx3cr1* knockout in CNS myeloid cells upregulated CXCL10 expression, which promoted immunosuppression and brain metastases of cancer cells originating from the lung, breast, colon, stomach, esophagus, pancreas, kidney, ovary, bladder, skin, and thyroid gland [40]. In brain metastases, indoleamine 2,3-dioxgenase-1 (IDO) appears to be a key regulator of autoimmunity, inhibiting T cell responses to apoptotic cell-associated antigens and controlling T cell activity at the site of graft-versus-host disease [45]. A recent study has reported that in melanoma brain metastases, comparative tissue analysis revealed microglia and tumor-associated macrophages as the primary sources of IDO expression, thus limiting T cell function in the brain parenchyma [46]. Similarly, intertumoral variations in IDO expression levels may mediate the surface expression of PD-L1 on T cells. Owing to the importance of IDO in tumor-mediated immunosuppression, small-molecule IDO inhibitors are currently in late-stage clinical trials as a strategy to enhance anti-tumor immunity [47]. *In vitro* co-culture has shown that collaboration between T cells and microglia leads to significant expression of immunosuppressive molecules, including IL-10, PD-1, and TGF-β [48, 49]. IL-10 increases the expression of TIM-3 on T cells [49]. Factors secreted by tumor cells, such as MIC-1, CSF-1, TGF-β, and IL-33, impair the anti-tumor functions of microglia. In addition, “don’t eat me” signals, such as CD47 and CD24, on glioma cells, which are potential ligands for SIRPα and Siglec-10 receptors on microglia, respectively, can impair the microglial phagocytosis [50]. We have schematically described microglial reprogramming and its roles in innate and adaptive immune responses in Figure 2.

Exosomes are 30-100 nm membrane vesicles that contain functional genetic material and proteins. Exosome-mediated intercellular communication greatly affects the brain microenvironment, contributing to the proliferation, survival, and colonization of cancer cells in the brain [51]. Exosomes are released by most cell types, including tumor cells and macroglia [52, 53]. Circulating tumor-derived exosomes not only predict metastatic tendencies, but also identify organ sites of future metastasis [54]. The dysregulation of miRNAs and proteins in breast cancer/melanoma cell line-derived exosomes promotes the adhesion and invasive properties of cancer cells in the brain [51]. Among them, upregulated miR-210, downregulated miR-19a and miR-29c, and elevated PDK1, 14-3-3ε, or receptors, such as AR, Erα, HER3, cyclin D1, and MAPK, are thought to promote brain metastases [51]. Deletion of XIST in breast cancer cells enhances the secretion of exosomal miRNA-503, which triggers the M1-M2 polarization switch in microglia, thereby upregulating immunosuppressive cytokines in microglia and suppressing T-cell proliferation [55]. Lung cancer cell-derived exosomes induce the release of endogenous Dkk-1 from brain endothelial cells, resulting in an absolute decrease in M1 microglia and a relative increase in M2 microglia, which acquire a pro-tumorigenic profile in the premetastatic niche [56]. The miR196a-5p released from the extracellular vesicles of nasopharyngeal carcinoma is transferred to microglia, which then regulates the structure and function of microglia by downregulating ROCK1 expression; promoting microglial proliferation, phagocytic activity, and inflammatory cytokine secretion; and inhibiting invasion of the brain parenchyma [57].

In addition to tumor-derived exosomes, exosomes from other cell types are substantially involved in brain metastasis. Astrocyte-derived exosomes mediate the intercellular transfer of PTEN-targeted miRNAs to metastatic tumor cells. Furthermore, the adaptive deletion of PTEN in brain metastatic tumor cells leads to increased secretion of chemokine CCL2, which recruits microglia expressing CCL2 receptor (CCR2) to mutually promote the growth of brain metastatic tumor cells by enhancing proliferation and reducing apoptosis [58].

### 2.3 Angiogenesis

Resident microglia are an alternative source of proangiogenic growth factors and cytokines and play a central role in the regulation of vascular homeostasis and angiogenesis in brain tumors [59]. Switching microglia from the M1 to the M2 phenotype is the first step in promoting angiogenesis. Peroxisome proliferator activated receptor-gamma (PPARγ) has been reported to be responsible for this conversion, and PPARγ agonists improve microglial polarization to the M2 phenotype [60-63]. After cerebral ischemia/reperfusion, M2 microglia promote neurogenesis and angiogenesis by secreting IGF-1, VEGF, and BDNF [64, 65]. Inhibition of angiopoietin-2 (Ang2)/VEGF reprograms pro-tumor M2 microglia toward an anti-tumor M1 phenotype [43].

In cancer research, tumor angiogenesis occurs at a late stage of metastasis after successful invasion and proliferation of cancer cells. M2 microglial polarization is positively correlated with microvessel density in patients with glioblastoma. M2 microglia express potent angiogenic factors, such as VEGF and CXCL2, to promote tumor growth. Blocking the CXCL2-CXCR2 signaling pathway is known to result in a significant reduction in glioma sizes [59]. Glioblastoma-associated M2 microglia promote the angiogenesis in glioblastoma via transporting the exosomal circKIF18A into human brain microvessel endothelial cells (hBMECs). Mechanistically, circKIF18A binds to and maintains FOXC2 stability and nuclear translocation in hBMECs [66]. Microglia also produce HGF/SF and express c-Met, which promotes angiogenesis by stimulating endothelial cell migration and proliferation [67-70]. Tumor-secreted S100 calcium-binding protein B (S100B) activates the receptor for advanced glycation end products (RAGE) on microglia, which induces STAT3 activation and suppresses the function of M1 microglia. Another study confirmed that activated RAGE signal in microglia maintains an M2-like phenotype and promotes glioma angiogenesis [71]. However, the role of microglia in promoting angiogenesis in extracerebral brain tumors remains largely unknown.

## 3. The crosstalk between microglia, astrocytes, and tumor cells promotes brain metastases

The ‘seed and soil’ hypothesis, first proposed by Stephen Paget, emphasizes the importance of reciprocal correspondence between tumor cells and host organs during the formation of metastatic lesions [72]. Metastasis to distant organs depends on the pathological crosstalk between tumor cells and various tissue-specific stromal components. Astrocytes and microglia are the two primary types of stromal cells in the brain [73]. Astrocytes and microglia reduce the number of metastatic brain cancer cells. Astrocytes are a part of the BBB because the end feet of astrocytes enclose the blood vessels [74]. Astrocytes and microglia can also be very hostile in terms of destroying metastatic cells. Most metastatic cancer cells do not survive because of the action of astrocytes, which produce plasmin from neuron-derived plasminogen and promote the release of membrane-bound astrocyte FasL. Another target of active plasmin, L1CAM, is an adhesion molecule that blocks the interactions between cancer cells and capillaries [75]. However, there is growing evidence that astrocytes and microglia may be “highjacked” by tumor cells for their settlement and development [76]. Reactive astrocytes have been shown to enhance tumor cell proliferation, survival, invasion, and resistance to chemotherapy [77, 78]. Microglia can be polarized into tumor-supportive and immunosuppressive cells by certain tumor-derived soluble factors, thus promoting tumor maintenance and progression.

The interaction between astrocytes and tumor cells is closely associated with brain metastases. Mechanistically, C-C motif chemokine ligand 2 (CCL2), produced mainly by astrocytes, promotes cancer cell chemotaxis through CCR2 [79]. Melanoma cells were found to alter the astrocyte secretion and evoke CCL2 expression and secretion, which in turn induced CCR2 expression in melanoma cells and enhanced their *in vitro* tumorigenic properties such as proliferation and metastasis [80]. Other proteins can also be released by astrocytes and tumor cells to establish brain metastases niche. Astrocytes also secrete neurotrophic factors such as the nerve growth factor (NGF) family of neurotrophins. Neurotrophin-3 (NT-3), a member of this family, promotes the growth of metastatic breast cancer cells in the brain by promoting their re-epithelialization and reducing the cytotoxic response of microglia [81]. Connexin43 (Cx43) protein, a major component of intercellular channels in astrocytes, promotes glioma cell migration and anti-apoptosis [82]. Metastatic tumor cells were also found to take advantage of Cx43 secretion to exchange nutrients and metabolites, thereby establishing brain metastases niche [76].

In addition to inflammatory, angiogenic, and immunomodulatory processes, interactions between cancer cells and microglia are directly involved in the proliferation and migration of cancer cells. Melanoma cells remodel microglia and upregulate matrix metalloproteinase-2 (MMP2) secretion to enhance cell proliferation and migration [83]. ANXA1 was found to be secreted by metastatic 4T1 mammary cancer cells and promoted microglial migration, which, in turn, promoted tumor cell migration. Silencing ANXA1 or inhibiting FPR1/FPR2 in 4T1 cells inhibited microglial migration and reduced STAT3 activation [84].

Furthermore, astrocytes and microglia cooperate with cancer cells at the same time to promote brain metastasis. miR-19a-containing exosomes are secreted by astrocytes and taken up by cancer cells to promote CCL2 expression. In turn, increased CCL2 on tumor cells recruits microglia to stimulate proliferation and inhibit apoptosis of metastatic brain cancer cells [85]. In addition, astrocytes in the brain are capable of promoting the metastatic transformation of circulating breast cancer cells and localizing them to the brain through secretion of chemokine CXCL12 [78]. Consistently, high expression of CCL2 or CXCL12 has been observed in patients with advanced (metastatic) breast cancer [86]. In addition, interactions between astrocytes and other immune cells, (e.g., granulocytes), promote brain metastasis [87]. The upregulated functional molecules secreted by microglia, astrocytes, and tumor cells that promote brain metastases are listed in Table 1.

**Table 1:** Upregulated functional molecules secreted by microglia, astrocytes, and tumor cells promote brain metastases.

Molecules	Cellular Source	Function	Reference
**MMP2**	M1 Microglia	Damage BBB and degrade the tight junctions between capillary endothelial cells	[97]
**IL-1β**		Damage BBB and intrigue CNS inflammation	[98, 99]
**IL-6**			[28]
**COX-2**			[97, 100]
**CXCL10**		Recruit PD-L1^+^ CNS-native myeloid cells and suppress T cell function	[40, 44]
**IDO**	M2 Microglia	Suppress T cell responses to apoptotic cell-associated antigens	[46]
**CXCL2**		Promote tumor angiogenesis	[59]
**VEGF**			[43, 59]
**TGF-β**		Promote tumor growth and induce immunosuppressive niche	[39]
**IL-10**			[40]
**CCL20**		Promote tumor progression and stemness	[38]
**IGF-1**			[44]
**miR-19a containing Exosomes**	Astrocytes	Increase CCL2 secretion by tumor cells	[51]
**Connexin43**		Promote metastatic cancer cell migration and resistance to apoptosis	[76, 82]
**CCL2**		Recruit microglia and inhibit cancer cell apoptosis	[79, 80, 85]
**ANXA1**	Breast cancer cells	Promote microglial migration and enhance tumor cell migration	[84]
**miRNA-503**		Trigger M1-M2 polarization conversion of microglia, upregulate immune suppressive cytokines, and suppress T cell proliferation	[55]

**Figure 3.**
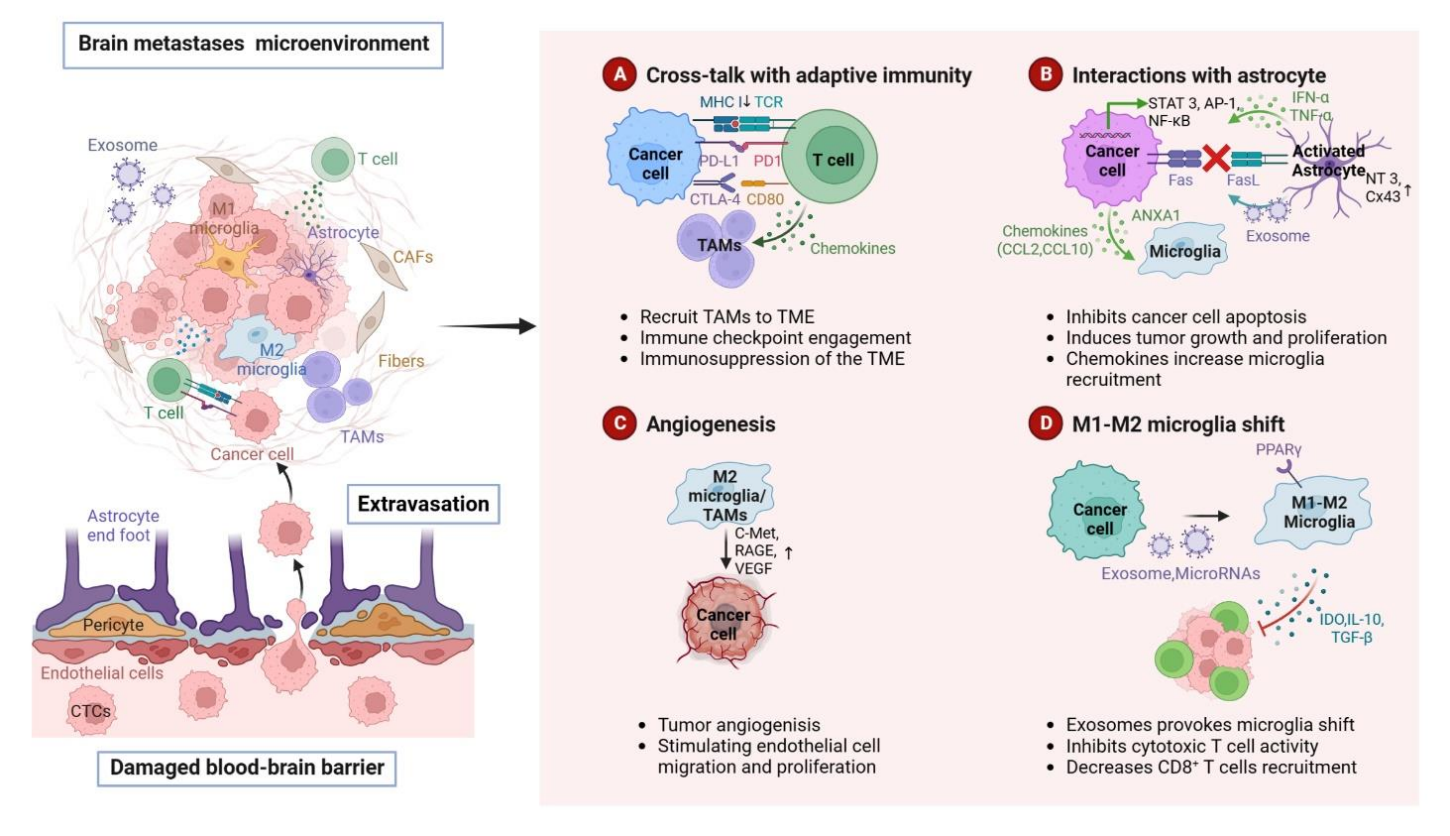
**Major mechanisms underlying brain metastasis**. Circulating tumor cells (CTCs) cross the blood-brain barrier (BBB) via extravasation after BBB disruption. Subsequently, the tumor microenvironment is reestablished through angiogenesis and reprogrammed immunity. Metastatic cancer cells produce molecules, such as miRNAs and immunosuppressive factors, that help them communicate and adapt to the brain environment. (**A**) Metastatic cancer cells reprogram adaptive immunity through the PD1/PD-l1 and CTLA4/CD80 immune checkpoints, leading to immune evasion and immunosuppression. (**B**) Astrocytes, cancer cells, and microglia interact with each other through the secretion of chemokines and cytokines. Activated STAT3, NF-κB, AKT-MAPK pathways in cancer cells lead to tumor proliferation and growth. CCL2 and CCL10, which are secreted by metastatic cancer cells, recruit more immunosuppressive microglia. (**C**) c-Met, RAGE, and VEGF overproduced by M2 microglia promote tumor angiogenesis. (**D**) Interactions between metastatic cancer cells, microglia, and T cells promote immune escape. Exosomal miRNAs contribute to microglial M2 polarization, which promotes cancer cell colonization and inhibits the cytotoxic effects of T-cells through the secretion of immunosuppressive cytokines.

## 4. Heterogeneous microenvironment of lung cancer and its reciprocal crosstalk with various stroma, immune cells, and extracellular matrix

Lung cancer is a highly heterogeneous disease. Cancer cells and cells within the tumor microenvironment (TME), including blood vessels, cancer-associated fibroblasts (CAFs), the extracellular matrix (ECM), and infiltrating immune cells, together determine the disease progression and response to therapy.

Immunosuppression is a major feature of the TME in lung cancer and is mediated by various immune cells. Lung cancer cells produce inhibitory molecules, including COX2, PGE2, PDL1, and IDO, which impair the activity of CD8^+^ tumor-infiltrating lymphocytes [88]. Dendritic cells (DCs) in patients with NSCLC upregulate the co-suppressor molecule B7-H3, and therefore fail to stimulate T cells [89]. Myeloid-derived suppressor cells (MDSCs), a heterogeneous cell population, inhibit T cell proliferation and cytokine production [90]. Neutrophil-infiltrating mouse tumors support tumor-associated inflammation, angiogenesis, and metastasis [91]. CAFs secrete IL-6, which stimulates JAK2-STAT3 signaling in human lung cancer cells and increases their metastatic potential [92]. Another study has reported that Gas6 from CAFs promotes the migration of Axl-expressing lung cancer cells during chemotherapy [93]. In addition, CAFs regulate immune responses. CAFs isolated from a subpopulation of human NSCLC cells expressing ligands of the PD1 receptor, namely PDL1 and PDL2, were shown to suppress T cell function [94]. The ECM, which is composed of collagens, proteoglycans, and glycosaminoglycans, is a major component of the TME and mediates the interactions between cancer and stromal cells to promote cancer progression. The receptor for the glycosaminoglycan hyaluronan, known as hyaluronan-mediated motility receptor (HMMR), enhances ECM-mediated signaling and facilitates the outgrowth of micrometastases [95]. Excessive collagen deposition in Lkb1-deficient lung tumors was found to lead to enhanced cancer cell proliferation and invasiveness through the activation of β1 integrin signaling [96].

We have schematically depicted the abovementioned main mechanisms underlying brain metastasis in Figure 3.

## 5. Microglia are a promising target for the treatment of metastatic brain cancers

### 5.1 Targeting anti-inflammatory microglia

Anti-inflammation in the CNS of the brain parenchyma helps in establishing an appropriate environment for tumor cell survival and colonization, and the induction of inflammation may inhibit brain metastasis [55, 58]. As mentioned above, microglia, as resident macrophages in the CNS parenchyma, play an important role in the onset and termination of inflammation, depending on their subtype [101].

The survival of resident microglia is dependent on CSF-1R signaling. PLX3397, a CSF1R inhibitor, blocked M2 polarization of microglia, which significantly suppressed nicotine-related brain metastases from lung cancer and prolonged brain metastasis-free survival [44]. Another CSF1R inhibitor, BLZ945, in combination with a STAT5 inhibitor, AC4-130, sustained tumor control and normalized microglial activation state [102]. PI3K signaling is a master regulator of metastasis-promoting microglia during CNS colonization. Buparlisib (BKM120), a pan-PI3K Class I inhibitor, re-educated microglia-induced invasion of breast cancer cells into the brain parenchyma [103]. In another study, exosomes were used to encapsulate curcumin (Exo-cur) or the signal transducer and STAT3 inhibitor JSI124 (Exo-JSI124). Intranasal administration of Exo-cur or Exo-JSI124 led to the rapid delivery of the exosome-encapsulated drug to the brain, selective uptake by microglia, and subsequent induction of microglial apoptosis. This strategy may provide a novel noninvasive therapeutic approach for the treatment of inflammation-related diseases in the brain [104].

### 5.2 Targeting brain immune metastatic niche and biomarkers of response to immune-checkpoint inhibitors

Toll-like receptors (TLRs) recognize a conserved set of molecular structures, known as pathogen-associated molecular patterns, which enable them to sense and initiate innate and adaptive immune responses. TLRs are expressed on microglia, neurons, astrocytes, and endothelial cells. Therefore, TLR agonists have received much attention as therapeutic agents against primary tumors and metastases [105, 106]. CpG-oligodeoxynucleotides (ODNs), which are TLR-9 agonists, enhanced antigen presentation by microglia and promoted apoptosis in glioma [107]. Another TLR-9 agonist, CpG-C, activated microglia to phagocytose tumor cells, thereby reducing brain metastasis from lung cancer and melanoma [108]. CpG oligodeoxynucleotides conjugated with carbon nanotubes (CNT-CpG) were found to be more potent than free CpGs. Intracranial melanomas were infiltrated by TLR-9 positive microglia, causing inflammatory responses and anti-tumor cytotoxicity against brain melanoma [109].

As mentioned above, melanoma-secreted Aβ impairs microglial phagocytosis. LY2886721, a BACE-i that blocks Aβ production, reduced the burden of brain metastases [41]. Parthenolide inhibited brain metastasis from lung cancer by blocking M2 polarization [44]. Elevated TGF-β secretion by microglia was detected in mice with intracerebral melanomas. However, blocking TGFβ signaling with small molecule inhibitors or monoclonal antibodies, namely 1D11 or LY2157299, did not improve their survival. In contrast, tumor antigen-specific vaccination combined with focal radiotherapy reversed the tolerance and improved survival. This regimen was found to be associated with enhanced CD8^+^ T cell polyfunctionality, increased ratio of T effector to T regulatory cells, and decreased microglial TGF-β release [110].

ICIs may be an effective treatment for brain metastases originating from melanoma; however, their clinical role in brain metastases from other solid malignancies remains uncertain [111]. Brain metastases are the most common type of brain tumors, harboring an immune microenvironment that can be targeted by immunotherapy [38]. ICIs enhance the immune recognition of tumors by interfering with CTLA-4, PD-1, LAG-3, Gal-9/TIM-3, and other pathways. Over the last decade, these agents have significantly improved the prognosis of patients with metastatic cancers [112]. Ipilimumab, with or without in combination with nivolumab and pembrolizumab, showed improved overall survival in patients with melanoma and brain metastases [3, 113, 114]. Another study has confirmed that the efficacy of these anti-PD-1 and anti-CTLA-4 inhibitors depends on the presence of extracranial disease and enhanced trafficking of CD8^+^ T cells into the brain [115]. The immunotherapeutic agents currently in clinical trials for the treatment of brain metastases are listed in Table 2.

The most widely studied predictive biomarkers for immunotherapy are PD-L1, microsatellite instability/ defective mismatch repair (MSI/dMMR), and tumor mutational burden (TMB). MSI/dMMR has been approved for clinical use irrespective of the tumor type, whereas PD-L1 has only been approved for certain cancer types (e.g., for predicting the response to first-line NSCLC pembrolizumab monotherapy). TMB can predict the responses to several immunotherapies in multiple cancer types; however, there is a lack of standardized assay system. Few studies have shown that tumor-infiltrating CD8^+^ lymphocytes, specific genetic signatures, and IDO1 and JAK mutations have the potential to predict immunotherapy responses [116-118]. New, efficient biomarkers with standardized assays and the role of microglia in predicting immunotherapy responses need to be investigated further.

**Table 2:** Ongoing clinical trials for immunotherapeutic agents for the treatment of brain metastases.

Interventions	NCT Identifier	Clinical Trial Phase	Cancer Types	Case Number	Primary End Point	Status	Ref.
**Sintilimab plus SRS**	NCT04180501	II	NSCLC	25	PFS	Not yet recruiting	[119]
**Camrelizumab plus WBR**	NCT04291092	II	NSCLC	63	PFS	Recruiting	[120]
**ICIs plus SRS**	NCT05522660	III	NSCLC, Melanoma	190	CNS-specific PFS	Recruiting	[121]
**Pembrolizumab plus SRS**	NCT02858869	II	NSCLC, Melanoma	27	Dose limiting toxicities	Not yet Recruiting	[122]
**Nivolumab and ipilimumab plus chemotherapy**	NCT05012254	II	NSCLC	71	PFS, ORR	Recruiting	[123]
**Pembrolizumab plus chemotherapy**	NCT04333004	I/II	NSCLC	40	iORR, iPFS, PFS	Unknown	[124]
**Tislelizumab plus carboplatin and pemetrexed**	NCT04507217	II	NSCLC	78	OS, PFS	Not yet recruiting	[125]
**Sintilimab plus bevacizumab**	NCT04213170	II	NSCLC	60	iORR	Unknown	[126]
**Camrelizumab plus pemetrexed and carboplatin**	NCT04211090	II	NSCLC	45	iORR	Active, not recruiting	[127]
**Pembrolizumab plus bevacizumab**	NCT02681549	II	NSCLC, melanoma	53	ORR	Recruiting	[128]
**Nivolumab plus SRS**	NCT02978404	II	NSCLC, SCLC	26	PFS	Active, not recruiting	[129]
**Durvalumab plus SRS**	NCT04889066	II	NSCLC	46	iDCR	Recruiting	[130]
**Nivolumab and radiotherapy with/without ipilimumab**	NCT02696993	I/II	NSCLC	88	PFS	Recruiting	[131]
**atezolizumab/tiragolumab/durvalumab**	NCT04513925	III	NSCLC	800	PFS	Recruiting	[132]

### 5.3 Targeting the re-programming of M1/M2 microglia

Microglia play a “friend or foe” role in brain metastasis, depending on the M1/M2 subtype upon activation. Therefore, reprogramming a pro-tumor/ immunosuppressive/anti-inflammatory M2 phenotype to an anti-tumor/immunostimulatory/inflammatory M1 phenotype is a strategy for combating tumor brain metastasis. Pharmacological inhibition of anti-inflammatory microglial phenotypes significantly reduced the tumor burden of breast cancer brain metastases [133]. Pretreatment with a phyto-glyceroglycolipid, 1,2-di-O-α-linolenoyl-3-O-β-galactopyranosyl-sn-glycerol (dLGG), drove the polarization of M2-like microglia to M1-like microglia and reduced melanoma brain metastases [134]. Tamoxifen treatment suppressed brain metastasis originating from breast cancer by blocking the polarization of M2 microglia and increasing their anti-tumor phagocytosis [36]. IL-6/JAK2/STAT3 signaling is involved in M2 microglial polarization. Tocilizumab, a monoclonal anti-IL6R neutralizing antibody, and fedratinib, a JAK2 inhibitor, were found to reduce the incidence of NSCLC brain metastasis [39]. We have described the promising microglial reprogramming targets in innate and adaptive immune responses in Figure 2.

### 5.4 Drug delivery systems

The poor prognosis of brain metastases is partially attributable to the BBB preventing most anticancer drugs from entering the brain [135]. Small molecules with improved BBB permeability are promising candidates in this regard [136]. Table 3 lists such inhibitors under clinical trials for the treatment of cancers with brain metastases. In addition, some large molecule monoclonal antibodies (mAbs) or antibody-coupled drugs (ADCs) have intracranial efficacy when the integrity of the BBB is compromised by the tumor itself, by radiotherapy, by low-intensity focused ultrasound (FUS) pulses, or with the assistance of delivery vehicles [137]. Studies pertaining to drugs with intracranial efficacy or adjuvant by delivery vehicles are essential for the treatment of brain tumors.

**Table 3:** Ongoing clinical trials for small molecular inhibitors for the treatment of brain metastases.

Compound	Target	NCT Identifier	Clinical Trial Phase	Cancer Type	Combination Partners	Ref.
**Osimertinib**	EGFR	NCT02296125	III	NSCLC with an EGFR mutation	None	[152]
**Aumolertinib**	EGFR	NCT03849768	III	NSCLC with EGFR Exon 19 deletion or L858R mutations	None	[153]
**Furmonertinib**	EGFR	NCT03787992	III	NSCLC with EGFRm	None	[154]
**MTI-31 (LXI-15029)**	mTOR	NCT03125746	I	Advanced malignant solid tumors	Exemestane	[140]
**T7-DSNPs/9291**	EGFR	NCT03257124	II	NSCLC with EGFR T790M	None	[150]
**Bevacizumab**	VEGF	NCT01004172	II	Breast cancer	carboplatin	[155]
**Trastuzumab**	HER2	NCT03529110	III	Breast cancer	None	[156]
**Trastuzumab**	HER2	NCT04752059	II	Breast cancer	None	[157]
**Tucatinib**	HER2	NCT02614794	III	Breast cancer	Capecitabine /Trastuzumab	[158]

Trifluoperazine (TFP), an antipsychotic agent, was found to suppress brain metastases from breast cancer and was highly bioavailable in the brain [138]. TFP has also been reported as a new adjuvant drug for treating patients with melanoma with brain, lung, and bone metastases, by disrupting the autophagic flux of melanoma [139]. MTI-31 (LXI-15029) is a novel mTORC1/mTORC2 inhibitor that is currently under clinical trials and has been found to reduce the recruitment of microglia in the TME [140].

Gold nanoparticles, superparamagnetic iron oxide nanoparticles, dual-targeting liposomes, and carbon dots have been suggested as delivery vehicles for treating brain metastases [141-144]. A microenvironment-tailored micelle (T-M/siRNA), co-delivering therapeutic siRNA and paclitaxel (PTX), could penetrate the BBB and target the immunosuppressive activation of microglia, thus significantly enhancing anti-tumor effects [145]. Mucic acid-based targeted nanoparticles, carrying camptothecin (CPT)/herceptin, significantly improved the therapeutic outcomes of brain metastases originating from breast cancer [146]. Hyaluronic-doxorubicin (hDOX), assembled using dual-targeting nanoparticles (NPs), showed the ability to reciprocally target the BBB and metastatic breast cancer cells by enzyme-recovered DNA insertion, thereby significantly prolonging the median survival time of mice [147]. Dual-targeting liposomal co-delivery systems provide another promising strategy for the treatment of patients with advanced EGFRT790M NSCLC and brain metastases, reducing the rate of drug resistance [148]. Lapatinib-loaded hiPSC platelets delivered to brain metastatic breast cancer cells inhibited the tumor growth and prolonged survival in tumor-bearing mice [149]. T7 peptide with osimertinib (AZD9291)-loaded intracellular glutathione (GSH)-responsive doxorubicin prodrug self-assembly nanocarriers (T7-DSNPs/9291) was designed as a potential targeted co-delivery system and exhibited anti-NSCLC brain metastasis effects [150]. CTLA-4 aldehyde modification was used as a CAR-targeting group to precisely target the central M1 microglia through aldehyde/hydroxylamine condensation on the surface of macrophages (CAR-M-UZPM) [151]. Notably, drugs targeting microglia, with or without delivery carriers, remain largely unexplored.

### Concluding remarks

Long-distance tumor-derived factors and local immune interactions are the main reasons for the establishment of a pre-metastatic niche in the brain. Different stimuli in the microenvironment determine the differentiation of microglia into an anti-tumor M1 phenotype or a pro-tumor M2 phenotype. Interactions between microglia and tumor cells, astrocytes, and other immune cells provide an appropriate environment for the survival and proliferation of metastatic tumor cells. In this review, we discussed the roles of microglia in inflammation, angiogenesis, and immune modulation. Therefore, targeting microglia is a potential therapeutic approach for treating metastatic brain tumors.

The attraction of ICIs lies in their ability to achieve long-term or even complete responses. However, most patients do not benefit from these treatments because of following biological and technical challenges, especially in the treatment of brain metastases:1) BBB impermeability, which hampers the entry of most molecules; 2) Tumor heterogeneity, which limits the efficacy of a single agent; 3) Expression of various immune checkpoints; 4) Lack of useful predictive biomarkers; And 5) Immune-related side effects. Therefore, it is important to study the small molecular inhibitors with intracranial permeability, the specific role of cellular components and their interactions in brain microenvironment. In addition, the combination of systemic therapy and stereotactic radiosurgery, or that of targeted therapy (anti-proliferating, pro-apoptotic, anti-angiogenic inhibitors) and ICIs, or dual ICIs (targeting different immune checkpoints) are potential strategies to treat brain metastases [3, 113, 114, 159, 160].

Recent studies have shown that metabolic regulation is closely associated with brain metastases. Brain-derived metabolism may provide a propensity for brain metastasis [161]. Further, Microglia polarization is associated with lactate metabolism and oxidative stress [162-164]. More studies pertaining to microglia-mediated metabolic re-reprogramming are needed. In addition, the role of microglia in the response to therapies remains unclear.
